# Reaching the London Declaration on Neglected Tropical Diseases Goals for Onchocerciasis: An Economic Evaluation of Increasing the Frequency of Ivermectin Treatment in Africa

**DOI:** 10.1093/cid/ciu467

**Published:** 2014-07-18

**Authors:** Hugo C. Turner, Martin Walker, Thomas S. Churcher, Mike Y. Osei-Atweneboana, Nana-Kwadwo Biritwum, Adrian Hopkins, Roger K. Prichard, María-Gloria Basáñez

**Affiliations:** 1 Department of Infectious Disease Epidemiology, School of Public Health, Faculty of Medicine (St. Mary's Campus), Imperial College London, United Kingdom; 2 Council for Scientific and Industrial Research, Water Research Institute; 3 Neglected Tropical Diseases Control Programme, Disease Control and Prevention Department, Ghana Health Service, Accra, Ghana; 4 Mectizan Donation Program, Decatur, Georgia; 5 Institute of Parasitology, Centre for Host–Parasite Interactions, McGill University (Macdonald Campus), Sainte Anne-de-Bellevue, Quebec, Canada

**Keywords:** cost effectiveness, economic evaluation, ivermectin, onchocerciasis, treatment frequency

## Abstract

***Background.*** Recently, there has been a shift in onchocerciasis control policy, changing from prevention of morbidity toward elimination of infection. Switching from annual to biannual ivermectin distribution may accelerate progress toward the elimination goals. However, the settings where this strategy would be cost effective in Africa have not been described.

***Methods.*** An onchocerciasis transmission framework (EpiOncho) was coupled to a disease model in order to explore the impact on disability-adjusted life years averted, program cost, and program duration of biannual ivermectin treatment in different epidemiological and programmatic scenarios in African savannah.

***Results.*** While biannual treatment yields only small additional health gains, its benefit is pronounced in the context of the elimination goals, shortening the time frames for and increasing the feasibility of reaching the proposed operational thresholds for stopping treatment. In settings with high precontrol endemicity (and/or poor coverage and compliance), it may not be possible to reach such thresholds even within 50 years of annual ivermectin, requiring adoption of biannual treatment. Our projections highlight the crucial role played by coverage and compliance in achieving the elimination goals.

***Conclusions.*** Biannual ivermectin treatment improves the chances of reaching the 2020/2025 elimination goals, potentially generating programmatic cost savings in settings with high precontrol endemicity. However, its benefit and cost are highly sensitive to levels of systematic noncompliance and, in many settings, it will lead to an increase in costs. Furthermore, it may not always be feasible to implement biannual treatment, particularly in hard-to-reach populations. This highlights the continued need for a macrofilaricide.

## Abstract (precis)

Although switching from annual to biannual ivermectin treatment yields small additional health benefits, in the context of elimination goals its benefit is pronounced, increasing the feasibility of and shortening the time frames for reaching proposed operational thresholds for stopping treatment.

Onchocerciasis, caused by the filarial nematode *Onchocerca volvulus*, is transmitted via bites of *Simulium* blackfly vectors [1], particularly *S. damnosum sensu lato* (s.l.) in Africa, where 99% of those at risk live. The adult worms, residing in nodules, produce microfilariae that migrate throughout the host's body [1]. Continuous exposure to microfilariae can lead to intense itching, skin lesions, visual impairment, blindness, and an increased risk of mortality [1–4].

The predominant onchocerciasis control strategy in Africa is community-directed annual mass drug administration (MDA) with ivermectin (Mectizan). Merck & Co. has committed to donate the drug for as long as needed to eliminate onchocerciasis as a public health problem [5]. Recently, there has been a shift in the onchocerciasis control policy in Africa, with the aim of programs changing from morbidity control to elimination of infection. The African Programme for Onchocerciasis Control (APOC) has a new goal of elimination of onchocerciasis where possible by 2025 [6], and the London Declaration on Neglected Tropical Diseases (LDNTD), on 31 January 2012 [7], joined the World Health Organization's (WHO) road map for accelerating work to overcome the global impact of neglected tropical diseases (NTDs). One of the proposed goals is the elimination of onchocerciasis in selected African countries by 2020 [8]. In this context, switching to biannual treatment in Africa might improve chances of elimination [9–11], a strategy partly motivated by its success in onchocerciasis foci in Latin America [10, 12] and used in several of the special intervention zones of the former Onchocerciasis Control Programme in West Africa (OCP), in particular, in the Mafou and Tinkisso basins of Guinea-Conakry and the Oti basin of Togo/Benin [13]. However, the likely impact of this strategy more generally in Africa and how it can help achieve the goals set by the WHO [8] has not been investigated.

Here, by linking a transmission dynamics and a disease model, we evaluate the health impact, programmatic cost, and projected duration of biannual vs annual ivermectin treatment in a range of endemic, economic, and programmatic scenarios typical of savannah onchocerciasis foci in Africa.

## METHODS

### Transmission Model

The analysis is underpinned by a host sex- and age-structured deterministic onchocerciasis transmission model (EpiOncho) [14, 15], parameterized for savannah areas [14] with perennial transmission, where the prevailing *O. volvulus–S. damnosum* s.l. combinations (ie, savannah parasites *S. damnosum sensu stricto* [s. str.]/*S. sirbanum*) are responsible for the most severe sequelae of the infection [1]. The underlying demography is that of northern Cameroon, assuming a stationary age distribution and a stable (closed) population [14]. The model has been modified to incorporate the temporal dynamics of the microfilaricidal and embryostatic effects of ivermectin [16] and to investigate the influence of treatment compliance separately from that of coverage [15]. It has been assumed that treatment efficacies against microfilariae and female worm fertility do not change with repeated rounds of treatment (ie, no decrease in sensitivity to ivermectin). To account for reported effects of repeated, long-term exposure to ivermectin on adult worms (antimacrofilarial action) [17], it is assumed that rates of microfilarial production by adult females are reduced cumulatively by 7% per ivermectin standard (150 µg/kg) dose [18] (compatible with the results of Gardon et al [19]). However, due to uncertainty as to the magnitude of this antimacrofilarial effect [15], we also explore a weaker (1%) and stronger effect (30% per dose, as assumed in the ONCHOSIM model [20]) in the sensitivity analysis. A detailed description of the model has been presented [15].

### Operational Thresholds for Treatment Interruption Followed by Surveillance

Based on experience in some foci in Mali and Senegal [21, 22], APOC has set what we henceforth refer to as operational thresholds for treatment interruption followed by surveillance (OTTIS). Namely, these are a microfilarial prevalence (by skin snipping) of <5% in all surveyed villages and <1% in 90% of such villages and fewer than 0.5 infective larvae per 1000 flies [23]. We assumed that when the modeled microfilarial prevalence (all ages) fell to <1.4% (the weighted mean of the 2 prevalence thresholds above), measured just before the next treatment round, the OTTIS would have been achieved, in turn, determining MDA program duration. Following the guidelines proposed by APOC, we focused on the microfilarial prevalence because we found the entomological threshold to be less useful in our projections, as this was consistently reached earlier than the microfilarial prevalence threshold and we used the more conservative indicator. It is important to realize that the OTTIS values are not truly a transmission breakpoint (parasite density below which the worm population would not be able to maintain itself [24]), but rather programmatic goals that indicate the cessation of MDA and the commencement of post-MDA surveillance. As OTTIS values are provisional [23], we vary them in the sensitivity analysis (Table 1).

**Table 1.** Summary of Parameter Definitions and Values Explored in the Sensitivity Analysis

Parameter	Value
Overall proportion of the total population receiving ivermectin at each round, referred to as therapeutic coverage (Note: This is the coverage of the total population and not of the eligible population as used by the Onchocerciasis Elimination Program for the Americas)	60%–80%
Proportion of the eligible population who never take treatment, referred to as the proportion of systematic noncompliers	0.1%–5%
Increase in cost (per year) of biannual compared with annual community-directed treatment with ivermectin	40%–80%
Discount rate applied to the health benefits and costs	0%–6%
Inclusion of the economic value of the donated ivermectin tablets	See *Methods*
The per dose reduction in microfilarial production of female adult worms, referred to as the antimacrofilarial action of ivermectin	1%–30%
Operational thresholds for treatment interruption followed by surveillance	1.4% ± 0.5% microfilarial prevalence

### Health Impact

Disability-adjusted life years (DALYs) averted were used to quantify the health impact of ivermectin, combining the burden of onchocercal disease resulting from blindness, visual impairment, troublesome itching, and premature death into a single metric [18]. The DALY burden was estimated using a disease model that links the dynamic transmission model–derived prevalence and intensity of *O. volvulus* infection with the burden of onchocercal disease [18].

### Cost of Mass Drug Administration

Based on cost data collected in savannah foci in Ghana [25], it was estimated that the economic cost of annual community-directed treatment with ivermectin (CDTI) is $41 536 per target population of 100 000 individuals (overall population) per year (2012 prices) and that this would increase by 60% when treating biannually [25]. However, due to uncertainty in generalizing this estimated cost increase to other African countries, this was varied in the sensitivity analysis. Costs were collected from the healthcare providers' perspective, that is, national control programs of endemic countries, nongovernment organization partners, and volunteer community distributors [25]. However, as part of the sensitivity analysis, we also included the additional economic value of donated ivermectin, assuming a commercial, per tablet, price of $1.50 plus $0.005 shipping costs, and that an average treatment requires 2.8 tablets per person [26].

### Model Outcomes and Sensitivity Analysis

The model was used to compare the impact of annual vs biannual CDTI over a 50-year time horizon in terms of the projected health gain (DALYs averted), program cost, and duration (Table 2). This long-time horizon was used in order to compare adequately the 2 strategies in the context of 2020/2025 elimination goals; MDA programs have been ongoing in many areas since the mid 1990s (in some areas, since 1988). Three precontrol endemicity levels, namely, 40%, 60%, and 80% precontrol microfilarial prevalence, were investigated to represent a range from mesoendemic to highly hyperendemic areas [27]. A summary of the precontrol conditions for the 3 endemicity levels investigated is shown in Supplementary Table 1. Changing to a biannual treatment strategy at different stages of an ongoing annual MDA program was also investigated; switching to twice-yearly CDTI at microfilarial prevalence values of 30%, 20%, and 15% (motivated by programmatic assessments conducted in Ghana before switching to biannual treatment in 2009 [25]). In line with WHO guidelines [28], we applied a discount rate of 3% to both the health benefits and the costs, and this rate was varied in the sensitivity analysis. Table 1 summarizes the parameter definitions and values that were explored in the sensitivity analysis.

**Table 2.** Outcome Metrics

**Ratio of health impact:** The ratio (biannual/annual) of the projected number of disability-adjusted life years (DALYs) averted by biannual vs annual mass drug administration (MDA). If this equals 1, biannual MDA has no additional health benefit over that of annual MDA. Values greater than 1 indicate benefit.
**Ratio of total cost:** The ratio (biannual/annual) of the total projected cost of biannual vs annual MDA. If this equals 1, biannual treatment costs the same as annual treatment. Values less than 1 indicate that biannual treatment generates cost savings compared with annual MDA.
**Ratio of the additional cost:** The ratio (biannual/annual) of the projected additional cost of biannual vs annual MDA considered from a point when an annual program switches to biannual treatment.
**Cost-effectiveness ratio of annual MDA:** The ratio between the projected total cost of annual MDA and the projected number of DALYs averted, that is, the cost per DALY averted. For example, if an intervention costs $100 and averts 5 DALYs, its cost-effectiveness ratio is 20. The lower this ratio, the more cost effective the intervention is considered to be.
**Incremental cost-effectiveness ratio of biannual MDA:** The ratio between the incremental cost of biannual treatment and the incremental number of DALYs averted (ie, over and above those costs and benefits of annual treatment) compared with annual MDA. This ratio measures the additional cost per additional health impact produced by using a biannual compared with annual MDA treatment strategy and evaluates whether the additional health benefits of an alternative intervention are worth the additional cost.

## RESULTS

**Figure 1.**
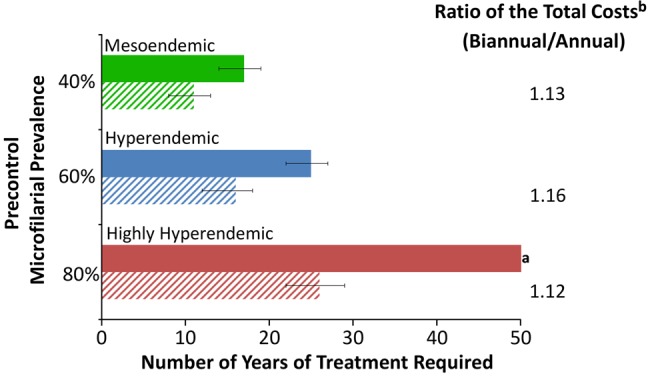
Comparison of annual vs biannual ivermectin treatment in areas where onchocerciasis control has not been previously implemented. Annual and biannual ivermectin treatments are indicated by solid and dashed bars, respectively. Error bars represent varying the operational thresholds for treatment interruption (1.4% microfilarial prevalence) by ±0.5%. The analysis was performed with a 50-year time horizon, discount rate of 3% applied both to costs and health benefits, therapeutic coverage of 80%, 0.1% systematic noncompliers, perennial transmission, and a 7% cumulative reduction in microfilarial production by female adult worms per ivermectin dose. A summary of the precontrol conditions is provided in Supplementary Table 1. See Table 2 for an explanation of terms. ^a^Operational threshold for treatment interruption not attained within the 50-year time horizon; ^b^Costs do not include those incurred by Merck & Co.

**Figure 2.**
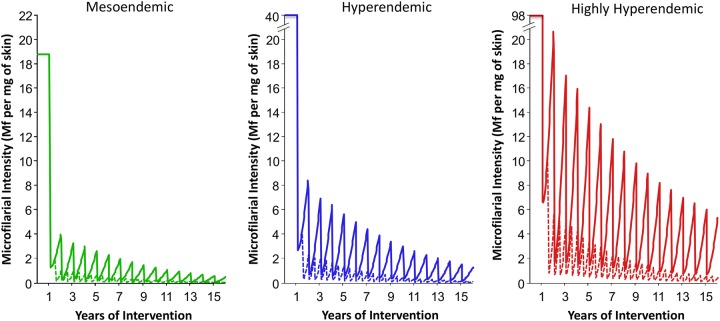
Comparison of the impact of annual and biannual ivermectin treatment on onchocercal microfilarial intensity. Annual and biannual ivermectin treatments are indicated by solid and dashed lines, respectively. The green, blue, and red lines correspond to a precontrol endemicity of 40%, 60%, and 80% microfilarial prevalence, respectively. Microfilarial intensity is quantified as the mean microfilarial load (Mf) per milligram of skin in those aged ≥20 years. The analysis was performed assuming a therapeutic coverage of 80%, 0.1% systematic noncompliers, perennial transmission, and a 7% cumulative reduction in microfilarial production by female adult worms per ivermectin dose.

Model outputs indicate that annual CDTI is highly cost effective (Table 3 and Supplementary Figure 1). The health impact, cost effectiveness, and projected MDA duration were strongly related to precontrol endemicity levels; the higher the initial microfilarial prevalence, the greater the health impact and cost effectiveness but the longer the projected program duration (Table 3 and Figures 1 and 2).

**Table 3.** Cost Effectiveness of Annual and Biannual Ivermectin Treatment Programs for Onchocerciasis Control at Different Levels of Precontrol Endemicity

Precontrol Endemicity (Microfilarial Prevalence)	Ratio of Total Health Impact (Biannual/Annual)	Ratio of Total Cost (Biannual/Annual)	Cost-Effectiveness Ratio of Annual Ivermectin Treatment ($)	Incremental Cost-Effectiveness Ratio of Biannual Ivermectin Treatment ($)
Mesoendemic (40%)	1.02	1.13	15^a^	100^b^
Hyperendemic (60%)	1.03	1.16	6^a^	36^a^
Highly hyperendemic (80%)	1.03	1.12	3^a^	12^a^

See Table 2 for an explanation of terms.

^a^ Highly cost effective (<$40 per DALY averted).

^b^ Cost effective ($40 to $238 per disability-adjusted life years [DALY] averted) based on the World Bank cost-effectiveness thresholds (inflated to their 2012 equivalent) [29]. The analysis was performed with a 50-year time horizon, discount rate of 3% applied both to costs and health benefits, therapeutic coverage of 80%, 0.1% systematic noncompliers, perennial transmission, and 7% cumulative reduction in microfilarial production by female adult worms per ivermectin dose. Costs do not include those incurred by Merck & Co. A summary of the precontrol conditions is provided in Supplementary Table 1. See Supplementary Table 2 for additional data.

**Figure 3.**
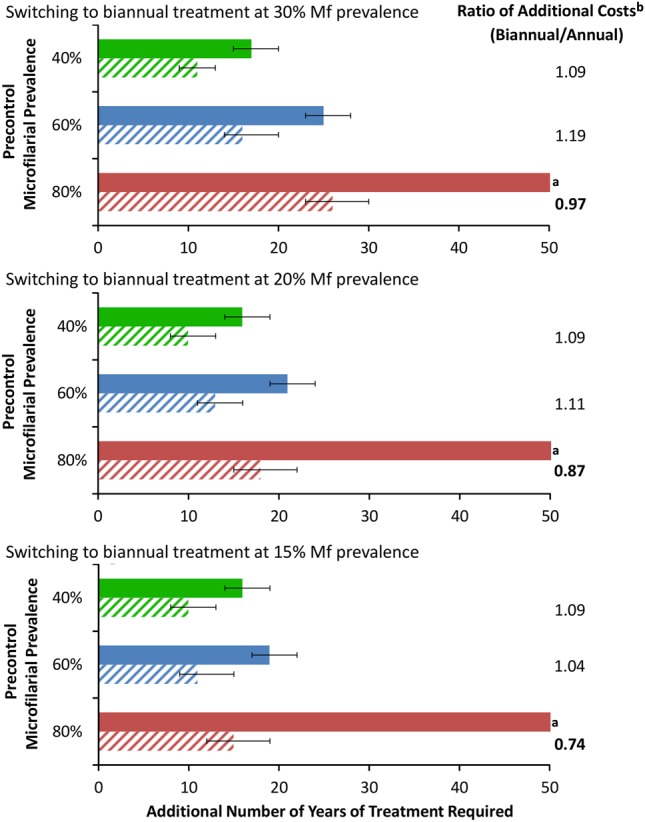
Impact of switching to biannual ivermectin treatment at different stages of an ongoing annual onchocerciasis treatment program. The green, blue, and red lines correspond to a precontrol endemicity of 40%, 60%, and 80% microfilarial (Mf) prevalence, respectively. Annual and biannual ivermectin treatments are indicted by solid and dashed bars, respectively. Error bars represent varying the operational thresholds for treatment interruption by ±0.5%. The number of additional years of treatment and the ratio of additional costs are considered from the point of the switch to biannual treatment (as opposed to the start of control). The microfilarial prevalence at the time of the switch was assumed to be measured just before the next round of treatment is distributed. The analysis was performed with a 50-year time horizon, discount rate of 3% applied both to costs and health benefits, therapeutic coverage of 80%, 0.1% systematic noncompliers, perennial transmission, and a 7% cumulative reduction in microfilarial production by female adult worms per ivermectin dose. See Table 2 for an explanation of terms. ^a^Operational threshold for treatment interruption not attained within the 50-year time horizon; ^b^Costs do not include those incurred by Merck & Co.

The projected incremental health gain of biannual vs annual CDTI (ie, the additional number of DALYs averted) was small, with biannual treatment not being more cost effective than annual treatment (Table 3). However, biannual treatment notably shortened duration of MDA. Additionally, switching from an annual to a biannual treatment strategy during an ongoing MDA program can also reduce program duration, particularly in highly hyperendemic areas (where annual CDTI would not suffice to reach OTTIS), potentially generating programmatic cost savings (Figure 3). In mesoendemic foci, the reduction in program duration was less pronounced. Furthermore, heterogeneity in the projected program duration among areas of different precontrol endemicity is substantially reduced when a biannual treatment strategy is used (Figure 3).

### Sensitivity Analysis

#### Therapeutic Coverage and Compliance

**Figure 4.**
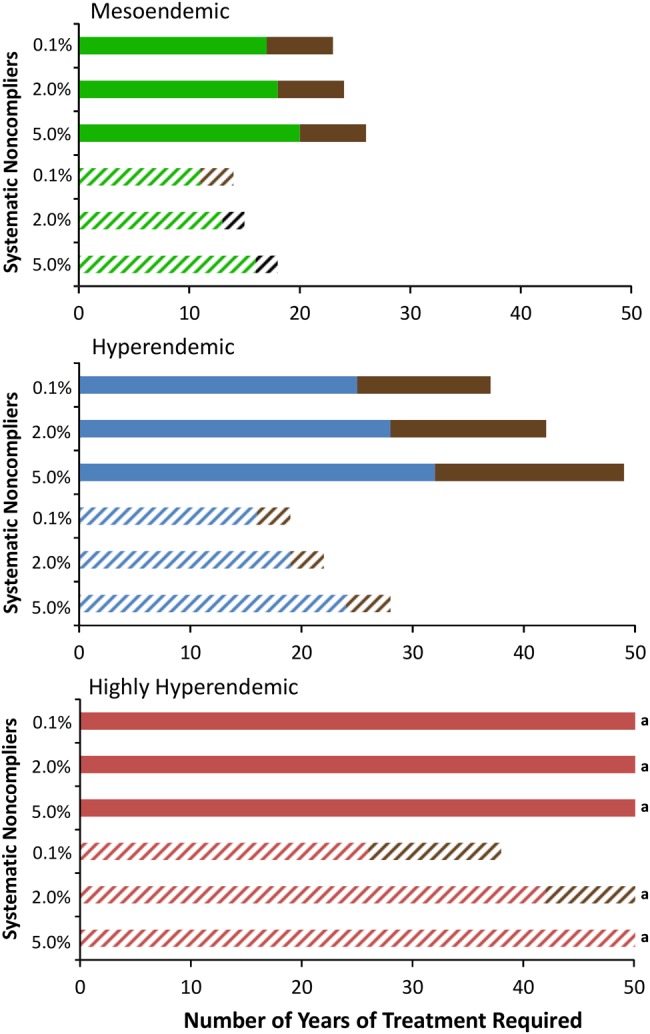
Sensitivity of the projected duration of annual and biannual ivermectin treatment programs for onchocerciasis control to different levels of coverage and systematic noncompliance. The green, blue, and red lines correspond to a precontrol endemicity of 40%, 60%, and 80% microfilarial prevalence, respectively. Dark brown bars represent the increment in program duration as a result of a decrease in the assumed therapeutic coverage from 80% to 60%. Annual and biannual ivermectin treatments are indicted by solid and dashed bars, respectively. The analysis was performed with a 50-year time horizon and a 7% cumulative reduction in microfilarial production by female adult worms per ivermectin dose. See Table 2 for an explanation of terms. ^a^Operational threshold not attained within the 50-year time horizon.

Varying the levels of therapeutic coverage and systematic noncompliance (Table 1) did not affect substantially the projected health impact of annual or biannual CDTI (Table 4). However, if the therapeutic coverage is low, there is a slightly greater incremental health gain when treating biannually (Table 5). Varying coverage and compliance markedly influenced the projected program duration and total cost. Therapeutic coverage exerted a more pronounced effect (which increased with increasing precontrol endemicity) on annual CDTI, while systematic noncompliance had a pronounced effect on biannual CDTI (Tables 4 and 5 and Figure 4).

**Table 4.** Sensitivity of Health Impact, Total Cost, and Duration of Annual and Biannual Ivermectin Treatment Programs for Onchocerciasis Control to Different Levels of Coverage and Systematic Noncompliance

Precontrol Endemicity	Percentage Change in Health Impact		Percentage Change in Total Cost		Percentage Change in Programme Duration	
	Annual	Biannual	Annual	Biannual	Annual	Biannual
Effect of assuming 60% vs 80% overall therapeutic coverage						
Mesoendemic	−4	−1	24	15	35	27
Hyperendemic	−4	−1	27	14	48	19
Highly hyperendemic	−3	−1	NA	25	NA	46
Effect of assuming 5% vs 0.1% systematic non-compliance						
Mesoendemic	−2	−2	13	35	18	45
Hyperendemic	−2	−3	17	34	28	50
Highly hyperendemic	−3	−4	NA	43	NA	285

See Table 2 for an explanation of terms. Precontrol microfilarial prevalence and modeling assumptions are as in the legend of Table 3.

Abbreviation: NA, operational thresholds for treatment interruption not attained within the 50-year time horizon.

**Table 5.** Sensitivity of the Relative Health Impact and Total Cost of Biannual Compared With Annual Ivermectin Treatment Programs for Onchocerciasis Control to Different Levels of Coverage and Systematic Noncompliance

Precontrol Endemicity	Systematic Non-compliance (%)	80% Overall Therapeutic Coverage		60% Overall Therapeutic Coverage	
		Ratio of Total Health Impact (Biannual/Annual)	Ratio of Total Cost (Biannual/Annual)	Ratio of Total Health Impact (Biannual/Annual)	Ratio of Total Cost (Biannual/Annual)
Mesoendemic	0.1	1.02	1.13	1.05	1.11
	5.0	1.02	1.35	1.05	1.24
Hyperendemic	0.1	1.03	1.16	1.06	1.04
	5.0	1.02	1.33	1.06	1.19
Highly hyperendemic	0.1	1.03	1.12	1.04	1.40
	5.0	1.02	1.60	1.05	1.60

Precontrol microfilarial prevalence and modeling assumptions are as in the legend of Table 3. See Table 2 for an explanation of terms.

#### Economic Assumptions

The incremental total cost of starting with, or switching to, biannual treatment was highly sensitive to the relative increase in the cost of biannual vs annual CDTI (Supplementary Table 3). Increasing the discount rate reduced the cost effectiveness of both annual and biannual CDTI, with this reduction being more pronounced the lower the precontrol endemicity level.

The cost effectiveness of both annual and biannual CDTI (and the potential cost savings) was substantially reduced by the inclusion of the economic value of the donated ivermectin tablets. However, the cost-effectiveness ratios of annual treatment remained under the World Bank thresholds for this strategy to be considered cost effective (Supplementary Tables 4 and 5) [29].

#### Ivermectin Antimacrofilarial Action

The magnitude of the assumed antimacrofilarial effect of ivermectin (on rates of microfilarial production by female worms) had little influence on health impact. When a negligible antimacrofilarial action was assumed, the projected duration for both strategies was longer, although biannual treatment still produced a notable reduction in duration (Supplementary Table 6). However, the larger the assumed effect, the shorter the projected duration of annual MDA (underscoring the desirability of having a truly macrofilaricidal drug, or a drug with a more profound effect on female worm fertility). This consequently decreased the incremental benefit (in terms of the reduction in program duration) of switching to biannual treatment, particularly in highly hyperendemic areas. Under greater antimacrofilarial action scenarios, biannual treatment would still considerably shorten projected program duration but would not generate programmatic cost savings (Supplementary Table 6).

## DISCUSSION

Our results suggest that annual CDTI has a large and highly cost-effective impact on human health. This is consistent with previous appraisals [26, 30, 31]. Reaching the operational thresholds suggested by APOC [23] in mesoendemic and borderline hyperendemic areas (those close to 60% microfilarial prevalence) is likely to be feasible for 2020/2025 using annual CDTI if coverage and compliance levels are high, which is in agreement with epidemiological observations [21, 22]. (However, these observations pertain to foci with seasonal [by *S. sirbanum*] as opposed to perennial transmission).

By contrast, our projections indicate that in initially highly hyperendemic areas (represented here by 80% microfilarial prevalence), it may not be feasible to reach the proposed operational thresholds with annual ivermectin treatment alone, even with high levels of coverage and compliance. This is because, in the absence of vector control, there is substantial transmission between consecutive annual treatments under scenarios of perennial transmission [18] (Figure 2). Although under these conditions, biannual ivermectin treatment would only have a small additional health impact—and would be deemed less cost effective than annual treatment in terms of the additional cost per additional DALY averted—it would lead to reduced program duration.

The impact of biannual treatment was strongly related to precontrol endemicity, with greater projected benefits for higher initial infection prevalence, greatly reducing the residual intertreatment transmission (Figure 2). In areas with lower precontrol endemicity (lower vector biting rates), such transmission becomes less important and biannual treatment has a lesser impact, yet still shortens program duration (Figure 1 and 2). Our projections also indicate a notable benefit of switching to biannual treatment during an ongoing annual MDA program (Figure 3). This is supported by a recent epidemiological study in the Abu Hamed focus of Sudan, which reported that switching from annual to biannual treatment from 2007 hastened interruption of transmission [32], as well as by reports of interruption of transmission in the Wadelai focus of northwest Uganda, where treatment frequency was increased to twice a year from 2006 [33]. This suggests that the true value of a biannual treatment strategy lies in its potential to accelerate progress toward reaching the elimination goals proposed by the LDNTD and WHO, instead of bringing additional health gains. Therefore, cost-effectiveness analysis (ie, the cost per DALY averted or health gain within a given time horizon) is not necessarily the most informative metric by which to judge biannual CDTI. This highlights the need for the development of further economic evaluation frameworks, which better account for the long-term benefits of elimination, to appraise more appropriately the potential impact of alternative treatment strategies for those NTDs targeted for elimination [8].

Although the current OTTIS used by APOC are supported by the epidemiological and entomological evaluations in Mali and Senegal [21, 22] and the experiences obtained during the OCP [23], they are provisional operational thresholds and are not necessarily equivalent to transmission breakpoints for elimination in all settings. Further validation and comparison of these OTTIS to true transmission breakpoints in different epidemiological settings—in the absence of vector control—is urgently needed. It should be noted that the WHO criteria for onchocerciasis elimination [34] (successfully used in Colombia [11, 12, 35] and the previously mentioned foci in Sudan and Uganda [32, 33]) recommend the demonstration of at least a 99% reduction in transmission potential and a 5-year cumulative incidence of fewer than 1 new case in 1000 sentinel group individuals during the 3-year period after treatment has been stopped (measured by Ov16 serology [36]). As the WHO transmission threshold [34] is considered relative to baseline, it accounts for potential differences in vector density, unlike the OTTIS entomological threshold, which is defined for a given number of flies (and therefore ignores the fact that even with a low number of L3 larvae per 1000 flies, the transmission potential can be considerable if the biting rate is high).

### Sensitivity Analysis

#### Coverage and Compliance

The health impact of ivermectin treatment was very robust across a range of different levels of therapeutic coverage and systematic noncompliance. Therapeutic coverage has a large bearing on the projected program duration and total cost of annual treatment, which is consistent with the results of other modeling studies [20, 23]. However, levels of systematic noncompliance have an even larger influence on the projected incremental cost and program duration of biannual MDA (Figure 4). This has important programmatic implications; in areas where there is low coverage but high compliance, biannual treatment may still provide benefit. This highlights the need to evaluate and understand the determinants of systematic noncompliance in programmatic evaluations [15]. The deleterious effects of low coverage and high systematic noncompliance increased in areas of high initial endemicity. In highly hyperendemic areas with low coverage and/or high systematic noncompliance, even a biannual treatment strategy may not be sufficient to reach the proposed OTTIS. Furthermore, even in mesoendemic/borderline hyperendemic areas with a low coverage and/or high systematic noncompliance, the 2020 goals set by the London Declaration/WHO are unlikely to be met [7, 8]. This highlights the importance of implementing or developing alternative or complementary intervention tools [37] such as vector control, macrofilaricidal therapies, more potent microfilaricides, and/or vaccines, as well as of conducting modeling studies to inform how best to combine these according to epidemiological and programmatic setting.

Our projections indicate that in communities with only moderate therapeutic coverage of annual CDTI (eg, 60%), efforts to increase the coverage to a higher level (eg, 80%) may have a similar (yet smaller) effect than increasing treatment frequency. However, we assumed the levels of therapeutic coverage and systematic noncompliance to be independent of treatment frequency. Yet it is conceivable that increasing treatment frequency to twice yearly may reduce systematic noncompliance and/or increase coverage because drug distribution would not always occur at the same time each year, with some individuals potentially being consistently missed due to seasonal work. In these circumstances, biannual treatment might have a larger impact than that presented here (provided sufficient efforts are made to maintain high coverage and compliance).

It should be noted that an assumed coverage in the total poplation of 80% would correspond to a very high coverage of the eligible population (see Table 1), which would be difficult to achieve operationally in many areas.

#### Economic Assumptions

The Ghana-specific estimate of a 60% increase in the cost (per year) of biannual vs annual CDTI (excluding the value of the donated drug) [25] is consistent with values for the increase in cost of biannual drug distribution for lymphatic filariasis control in Africa [38]. However, this cost will undoubtedly vary among countries and programmatic scenarios. Our sensitivity analysis illustrates that it has a large effect on the incremental cost of implementing from the start, or switching to biannual treatment. This highlights the need for countries that are considering changing to biannual treatment to assess the potential cost increase for their specific situation and other coendemic infections.

Despite the inclusion of the large economic value of the donated ivermectin tablets, annual CDTI remained cost effective, although such inclusion did raise the incremental cost of biannual treatment (Supplementary Tables 4 and 5). Further examination of other potential costs associated with increasing treatment frequency incurred by Merck & Co. is necessary, such as those of establishing new production lines to meet higher demands for ivermectin tablets.

#### Ivermectin Antimacrofilarial Action

The magnitude of the assumed antimacrofilarial effect of ivermectin (on rates of microfilarial production by female worms) had little influence on health impact but a greater one on program duration. When assuming a negligible antimacrofilarial action, the projected duration for both strategies became longer, although biannual treatment still produced a marked reduction in duration (Supplementary Table 6). The greater the assumed antimacrofilarial effect, the shorter the projected duration of annual MDA (underscoring the desirability of a truly macrofilaricidal drug, or a drug with a more profound effect on female worm fertility). This consequently decreased the incremental benefit (in terms of the reduction in program duration) of switching to biannual treatment, particularly in highly hyperendemic areas. Under greater antimacrofilarial action scenarios, biannual treatment would still considerably shorten projected program duration but would not necessarily generate programmatic cost savings (Supplementary Table 6).

### Potential Limitations and Other Considerations

Currently, EpiOncho is parameterized for savannah areas of Africa [14]. Consequently, conclusions are not necessarily directly generalizable to forest settings, which have different relationships between infection and sequelae [1, 39] and where onchocerciasis vectors are different members of the *S. damnosum* s.l. complex [40]. (This issue is presently being addressed but is outside the scope of the work presented here). Additionally, the disease burden associated with disfiguring skin lesions such as leopard skin was not quantified, and therefore the overall health impact of CDTI may be underestimated [18].

A fundamental assumption of our model is that of closed populations; there is no cross transmission or “spill over” infection between contiguous or otherwise proximate onchocerciasis foci. In reality, this is seldom the case; in some areas, treatment cannot be stopped due to the threat of reintroduction of infection from nearby areas where transmission is more intense, requiring more frequent or longer MDA. This would incur a cost that is not captured in this study. Consequently, the true programmatic value of the potential for biannual treatment to reduce heterogeneity in program duration among areas with different infection endemicities (different transmission intensities) is likely to be considerably underestimated. Furthermore, our analysis is performed within a 50-year time horizon; therefore, the true cost of having to continue annual CDTI beyond this point, particularly in highly hyperendemic areas, is not captured. Consequently, the potential cost savings generated by biannual CDTI are also underestimated.

Furthermore, it was implicitly assumed that onchocerciasis control is conducted independently from other control programs. However, onchocerciasis and lymphatic filariasis control activities are often carried out simultaneously. The possible implications of this on program costs, drug supplies, donation programs, and duration of drug distribution were not considered in this analysis. For instance, if MDA frequency were increased for lymphatic filariasis control, it may reduce the relative increase in cost of biannual CDTI for onchocerciasis.

Additionally, our analysis assumed that ivermectin's efficacy remained unchanged for the entire duration of the MDA programs and did not decrease due to development of ivermectin resistance. Biannual ivermectin treatment could have an even greater benefit in areas where suboptimal/atypical responses to ivermectin have been reported (such as in several Ghanaian communities [41, 42]). However, the overall benefit of biannual treatment in these circumstances will depend on the underlying causes of this phenomenon. (If it were due to genetic changes in the parasite population, increased treatment frequency could potentially impose a greater selection pressure and lead to decreased ivermectin efficacy).

### Conclusions and Implications for Onchocerciasis Control and Elimination

Biannual ivermectin treatment yields only small additional health benefits over those of annual treatment. However, in the context of elimination goals, the benefit of biannual treatment is pronounced, shortening time frames to reach proposed operational thresholds in the 2020/2025 time frames. This applies both to scenarios that deploy the biannual strategy from the outset or switch from an existing annual strategy. This effect becomes more pronounced for settings with high preintervention endemicity; in highly hyperendemic areas, reaching such thresholds would only be possible using biannual CDTI, provided therapeutic coverage and compliance are high. A biannual treatment strategy also homogenizes projected program duration among different initial endemicity settings and could act to mitigate cross transmission among contiguous onchocerciasis foci, as well as to reduce infection reintroduction into controlled areas. Reductions in program duration could potentially lead to programmatic cost savings. Projected outputs depend on assumptions of effects of prolonged ivermectin treatment on adult worms, coverage, compliance, and association between infection and disease.

In addition to cost, shorter programs are more attractive to donors, health officials, and politicians and are at a lower risk of disruption by economic and political instability. Notwithstanding these conclusions, the feasibility of increasing from 1 to 2 treatments yearly will vary with the specific programmatic circumstances of the country, availability of resources, and incremental cost. The benefit and cost of biannual treatment are particularly sensitive to levels of systematic noncompliance (ie, the proportion of the eligible population who never take treatment). This highlights the necessity for programs to strive for high compliance (not just coverage) and the need for the determinants and current levels of systematic noncompliance to be investigated [15].

## Supplementary Data

Supplementary materials are available at *Clinical Infectious Diseases* online (http://cid.oxfordjournals.org). Supplementary materials consist of data provided by the author that are published to benefit the reader. The posted materials are not copyedited. The contents of all supplementary data are the sole responsibility of the authors. Questions or messages regarding errors should be addressed to the author.

Supplementary Data
