# Supplementary material for: Reaching the London Declaration on Neglected Tropical Diseases Goals for Onchocerciasis: An Economic Evaluation of Increasing the Frequency of Ivermectin Treatment in Africa
Source: Clin Infect Dis. 2014 Jul 18;59(7):923–32. doi: 10.1093/cid/ciu467 (PMC4166981; doi:10.1093/cid/ciu467)
Supplement: Supplementary Data [file supp_59_7_923__index.html]

Reaching the London Declaration on Neglected Tropical Diseases goals for onchocerciasis: an economic evaluation of increasing the frequency of ivermectin treatment in Africa — Reaching the London Declaration on Neglected Tropical Diseases Goals for Onchocerciasis: An Economic Evaluation of Increasing the Frequency of Ivermectin Treatment in Africa — Reaching the London Declaration on Neglected Tropical Diseases Goals for Onchocerciasis: An Economic Evaluation of Increasing the Frequency of Ivermectin Treatment in Africa — Supplementary Data 

# Reaching the London Declaration on Neglected Tropical Diseases Goals for Onchocerciasis: An Economic Evaluation of Increasing the Frequency of Ivermectin Treatment in Africa

## Supplementary Data

Supplementary Data

**Files in this Data Supplement:**

- Supplementary Data - Pdf file
