# Supplementary material for: Reaching the London Declaration on Neglected Tropical Diseases Goals for Onchocerciasis: An Economic Evaluation of Increasing the Frequency of Ivermectin Treatment in Africa
Source: Clin Infect Dis. 2014 Jul 18;59(7):923–32. doi: 10.1093/cid/ciu467 (PMC4166981; doi:10.1093/cid/ciu467)
Supplement: Supplementary Data [file supp_ciu467_ciu467supp.pdf]

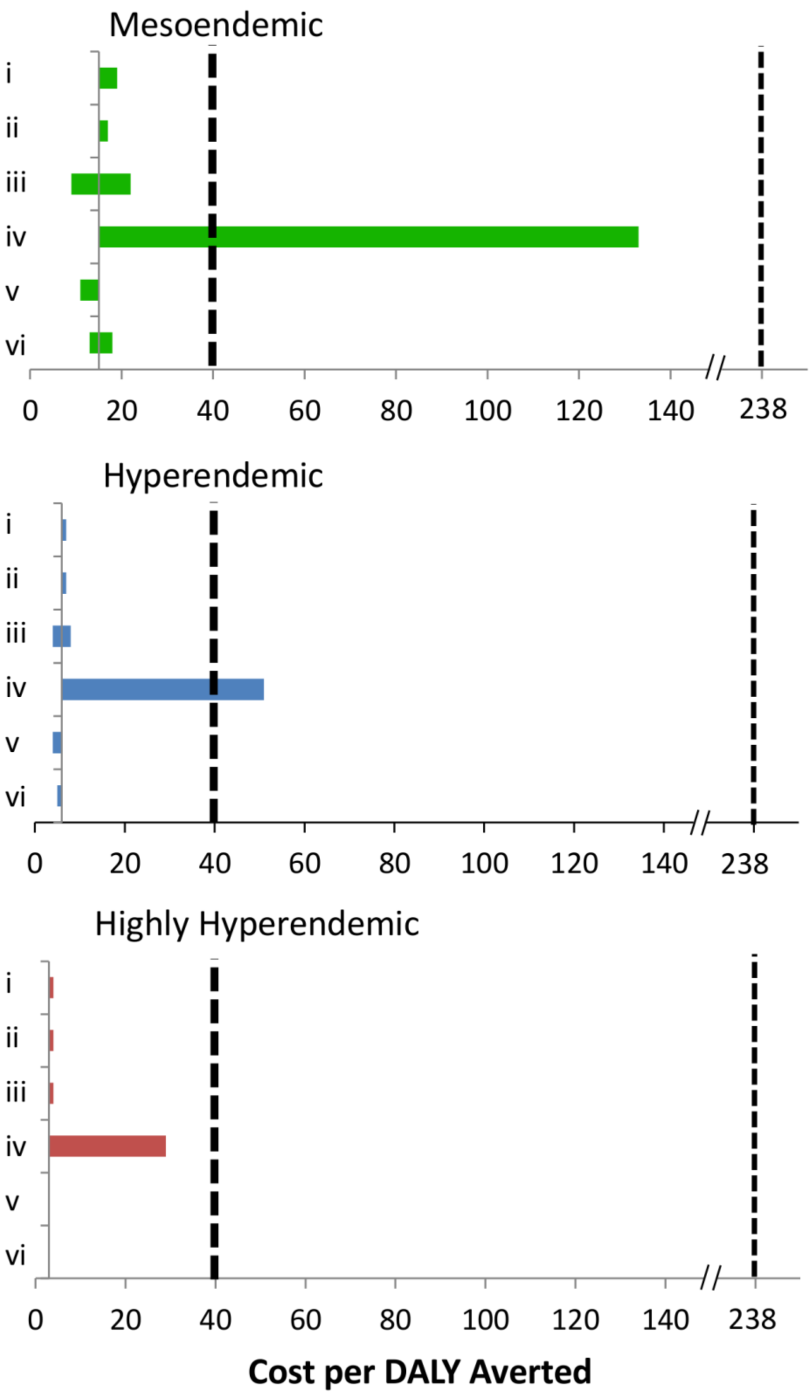

Supplementary Figure 1: Univariate sensitivity analysis of the cost-effectiveness of an annual ivermectin treatment programme for onchocerciasis control. The green, blue and red lines correspond to, respectively, a pre-control endemicity of 40%, 60%, and 80% microfilarial prevalence. The baseline cost-effectiveness (with the assumptions outlined in the legend of Figure 1) is indicated by the thin grey horizontal line. i: Decrease in therapeutic coverage from 80% to 60%. ii: Increase in the proportion of systematic non-compliance from 0.1% to 5%. iii: Change in the discount rate from 3% ± 3% (i.e. 0-6%). iv: Inclusion of the value of the donated ivermectin tablets. v: Higher anti-macrofilarial action of ivermectin (i.e. a 30% instead of a 7% per dose reduction in microfilarial production of exposed female adult worms). vi: Different operational thresholds for treatment interruption (1.4 ± 0.5%). Thick and thin dashed lines represent the thresholds for the intervention being highly cost effective (<USD 40 per DALY averted), and cost effective (<USD 238 per DALY averted), based on World Bank criteria of cost per DALY averted (inflated to their 2012 equivalent). USD: US Dollars.

Supplementary Table 1: Summary of pre-control conditions

Pre-control endemicity	Microfilarial prevalence	Annual biting rate§†	Annual transmission potential¶†	Mean intensity* (mf per mg)	Mean intensity* in those aged ≥ 20 (mf per mg)
Mesoendemic	40%	7,305	88	11.2	18.7
Hyperendemic	60%	15,472	373	23.9	40.0
Highly hyperendemic	80%	85,800	4,290	58.9	98.0

§ Annual biting rate (ABR): the average number of *Simulium* bites to which a person is exposed during a whole year.

¶ Annual transmission potential (ATP): the average number of infective larvae (L3) of *Onchocerca volvulus* potentially received during a whole year by a person exposed to the annual biting rate.

† Both the ABR and ATP are for a proportion of vector blood meals of human origin equal to 0.3[1].

* Arithmetic mean microfilarial load per mg of skin; note that this is different to the community microfilarial load (CMFL), which is the geometric mean microfilarial load per skin snip in those aged 20 years and above) [2].

Supplementary Table 2: The health impact and cost of annual and biannual ivermectin treatment programmes for onchocerciasis control at different levels of pre-control endemicity

Pre-control endemicity	Total cost (USD) of annual treatment (per 100,000)	Incremental cost (USD) of biannual treatment (per 100,000)	Total number of DALYs averted with annual treatment (per 100,000)	Incremental DALYs averted with biannual treatment (per 100,000)
Mesoendemic	554,049	72,346	37,858	727
Hyperendemic	729,357	117,006	129,884	3,258
Highly hyperendemic	1,067,5558	129,910	331,632	10,597

USD: US Dollars. The analysis was performed with a 50-year time horizon (and 100,000 individuals), discount rate of 3% applied both to costs and health benefits, therapeutic coverage of 80%, 0.1% systematic non-compliers, perennial transmission, and a 7% cumulative reduction in microfilarial production by female adult worms per ivermectin dose. Costs do not include those incurred by Merck & Co. A summary of the pre-control conditions is provided in Supplementary Table 1.

Supplementary Table 3: Sensitivity of the total cost of biannual compared to annual treatment programmes for onchocerciasis control to an increase in the yearly cost of biannual community-directed treatment with ivermectin

**Schedule of biannual ivermectin treatment strategy**	**Pre-control endemicity**	**Increase in cost (per year) of biannual with respect to annual community-directed treatment with ivermectin**	
		40%	80%
**Biannual ivermectin treatment implemented from start of the programme**		**Ratio of the total costs (biannual/annual)**	
	Mesoendemic	0.99	1.27
	Hyperendemic	1.02	1.31
	Highly hyperendemic	0.98	1.26
**Switching to biannual treatment at different levels of microfilarial prevalence in an ongoing annual treatment programme**		**Ratio of the additional total costs* (biannual/annual)**	
30% microfilarial prevalence	Mesoendemic	0.99	1.27
	Hyperendemic	1.04	1.34
	Highly hyperendemic	0.85	1.09
20% microfilarial prevalence	Mesoendemic	0.95	1.23
	Hyperendemic	0.97	1.25
	Highly hyperendemic	0.76	0.97
15% microfilarial prevalence	Mesoendemic	0.95	1.23
	Hyperendemic	0.91	1.17
	Highly hyperendemic	0.66	0.85

* The ratio of additional costs is considered from the point of switching from annual to biannual treatment (as opposed to from the start of control).When switching from annual to biannual treatment, infection (microfilarial) prevalence was assumed to be measured at the beginning of the programmatic year (i.e. just before treatment is distributed). Pre-control microfilarial prevalence and modelling assumptions are as in the legend of Supplementary Table 2.

Supplementary Table 4: Sensitivity of the cost-effectiveness of annual and biannual ivermectin treatment programmes for onchocerciasis control to the discount rate, and the economic value of the donated ivermectin

Pre-control endemicity	Cost-effectiveness ratio of annual ivermectin treatment (USD)†			Incremental cost-effectiveness ratio of biannual ivermectin treatment (USD) §		
**Excluding the value of (donated) ivermectin tablets**						
	**Discount rate**			**Discount rate**		
	0%	3%	6%	0%	3%	6%
Mesoendemic	9**	15**	22**	27**	100*	177*
Hyperendemic	4**	6**	8**	5**	6**	68*
Highly hyperendemic	3**	3**	4**	2**	12**	42*
**Including the value of (donated) ivermectin tablets**						
	**Discount rate**			**Discount rate**		
	0%	3%	6%	0%	3%	6%
Mesoendemic	79*	133*	197*	1,745	2,674	3,661
Hyperendemic	34**	51*	69*	477	859	1,257
Highly hyperendemic	26**	29**	33*	17**	334	711

USD: US Dollars. † The ratio of the total cost and the total number of DALYs averted (i.e. the cost per DALY averted) of an annual ivermectin treatment programme. § The ratio of the incremental cost and the incremental number of DALYs averted by a biannual compared to annual ivermectin treatment programme (i.e. the extra cost per extra health gain). ** Highly cost-effective (<USD 40 per DALY averted), * cost-effective (USD 40 to USD 238 per DALY averted) based on the World Bank cost-effectiveness thresholds (inflated to their 2012 equivalent) [3]. Pre-control microfilarial prevalence and modelling assumptions are as in the legend of Supplementary Table 2.

Supplementary Table 5: Sensitivity of the relative total cost of biannual compared to annual treatment programmes for onchocerciasis control to the discount rate

**Schedule of biannual ivermectin treatment strategy and initial level of onchocerciasis endemicity**		**Ratio of total cost (biannual/annual)**			**Ratio of total cost (biannual/annual)**		
		Excluding the value (donated) of ivermectin tablets			Including the value (donated) of ivermectin tablets		
**Biannual ivermectin treatment implemented from start of the programme**		**Discount rate**			**Discount rate**		
		0%	3%	6%	0%	3%	6%
	Mesoendemic	1.04	1.13	1.22	1.27	1.38	1.49
	Hyperendemic	1.03	1.16	1.28	1.26	1.42	1.57
	Highly hyperendemic	0.83	1.12	1.34	1.02	1.37	1.64
**Switching to biannual treatment at different levels of microfilarial prevalence in an ongoing annual treatment programme**		**Discount rate**			**Discount rate**		
		0%	3%	6%	0%	3%	6%
30% microfilarial prevalence	Mesoendemic	1.04	1.13	1.22	1.27	1.38	1.49
	Hyperendemic	1.07	1.19	1.30	1.31	1.46	1.59
	Highly hyperendemic	0.67	0.97	1.22	0.82	1.19	1.49
20% microfilarial prevalence	Mesoendemic	1.00	1.09	1.18	1.23	1.34	1.44
	Hyperendemic	0.99	1.11	1.22	1.22	1.36	1.49
	Highly hyperendemic	0.58	0.87	1.13	0.71	1.06	1.38
15% microfilarial prevalence	Mesoendemic	1.00	1.09	1.18	1.23	1.34	1.44
	Hyperendemic	0.93	1.04	1.14	1.14	1.27	1.40
	Highly hyperendemic	0.48	0.75	1.02	0.59	0.92	1.24

Pre-control microfilarial prevalence and modelling assumptions are as in the legend of Supplementary Table 2

Supplementary Table 6: Sensitivity of the health impact, total cost and duration of annual and biannual ivermectin treatment programmes for onchocerciasis control to the magnitude of the anti-macrofilarial action of ivermectin

		**1% cumulative reduction in microfilarial production by female adult worms per ivermectin dose**				**30% cumulative reduction in microfilarial production by female adult worms per ivermectin dose**			
**Schedule of ivermectin treatment strategy and initial level of onchocerciasis endemicity**		**Ratio total of health impact (biannual/ annual)**	**Ratio total of costs (biannual/ annual)**	**Projected duration of treatment programme (years)**		**Ratio total of health impact (biannual/ annual)**	**Ratio total of costs (biannual/ annual)**	**Projected duration of treatment programme (years)**	
				Annual frequency	Biannual frequency			Annual frequency	Biannual frequency
**Annual or biannual ivermectin treatment implemented from start of the programme**									
	Mesoendemic	1.02	1.18	21	14	1.01	1.26	12	9
	Hyperendemic	1.02	1.15	33	20	1.02	1.38	17	14
	Highly hyperendemic	1.02	1.40	50+	38	1.02	1.14	38	22
**Switching to biannual treatment at different levels of microfilarial prevalence in an ongoing annual treatment programme**									
30% microfilarial prevalence	Mesoendemic	1.02	1.15	20	13	1.01	1.22	11	8
	Hyperendemic	1.02	1.13	32	19	1.02	1.44	16	14
	Highly hyperendemic	1.02	1.25	50+	31	1.02	1.09	34	19
20% microfilarial prevalence	Mesoendemic	1.02	1.15	20	13	1.01	1.22	11	8
	Hyperendemic	1.02	1.10	29	17	1.01	1.43	15	13
	Highly hyperendemic	1.02	1.12	50+	26	1.02	1.09	32	18
15% microfilarial prevalence	Mesoendemic	1.02	1.15	20	13	1.01	1.22	11	8
	Hyperendemic	1.02	1.04	25	14	1.01	1.39	13	11
	Highly hyperendemic	1.02	1.00	50+	22	1.02	1.08	30	17

The analysis was performed with a 50-year time horizon, discount rate of 3% applied both to costs and health benefits, therapeutic coverage of 80%, 0.1% systematic non-compliers, perennial transmission, and. Costs do not include those incurred by Merck & Co.

References

1. Basáñez MG, Boussinesq M. Population biology of human onchocerciasis. Philos Trans R Soc Lond B Biol Sci **1999**; 354(1384): 809-26.
2. Remme JHF, Ba O, Dadzie KY, Karam M. A force-of-infection model for onchocerciasis and its applications in the epidemiological evaluation of the Onchocerciasis Control Programme in the Volta River basin area. Bull World Health Organ **1986**; 64(5): 667-81.
3. World Bank. World development report 1993: investing in health. New York: Oxford University Press**, 1993**. Available at: http://files.dcp2.org/pdf/WorldDevelopmentReport1993.pdf. Accessed 6 November 2013.
